# Work Ability during the Return to Work Process: Results from a Mixed Methods Follow-Up Study Among Employees with Common Mental Disorders

**DOI:** 10.1007/s10926-024-10262-3

**Published:** 2025-02-06

**Authors:** Alexandra Sikora, Ralf Stegmann, Ute B. Schröder, Inga L. Schulz, Uta Wegewitz, Ute Bültmann

**Affiliations:** 1 Division 3 Work and Health, Unit Evidence-Based Occupational Health, Workplace Health Management, Federal Institute for Occupational Safety and Health (BAuA), Nöldnerstr. 40-42, 10317, Berlin, Germany; 2 Department of Health Sciences, Community and Occupational Medicine, University of Groningen, University Medical Center Groningen, Groningen, The Netherlands

**Keywords:** Work ability, Return to work, Common mental disorders, Long-term sickness absence, Mixed methods

## Abstract

**Purpose:**

A main goal during the return to work (RTW) process after a long-term sickness absence due to common mental disorders (CMDs), is to restore and maintain employees’ work ability to enable their sustained work participation. This study jointly examined employees’ work ability ratings and experiences during their RTW process with CMDs.

**Methods:**

In a mixed methods follow-up study of *N* = 286 participants, work ability was quantitatively assessed with the Work Ability Score (WAS, range 0–10) at baseline (week before clinical discharge) and after 6, 12, 18, and 30 months. In a sub-sample, the qualitative work ability experiences of *N* = 32 participants were analysed at 6 and 12 months, and were jointly evaluated with the quantitative data.

**Results:**

The mean WAS increased during the first 18 months of follow-up. Three groups of qualitative work ability experiences emerged: Employees with (1) *poor work ability* (WAS 0–3), who did not RTW yet and described great difficulties in coping with everyday life, (2) *moderate work ability* (WAS 4–6), who mainly did RTW, but still showed a certain level of fragility, and (3) *good to very good work ability* (WAS 7–10), who mainly returned to work and reported many individual and work accommodations to maintain their work ability.

**Conclusion:**

The present study provides new insights into different aspects of work ability experiences, especially during the later RTW phases, where restoring and maintaining work ability is essential for a sustained work participation. This knowledge may help RTW stakeholders to better tailor support during the RTW process.

**Supplementary Information:**

The online version contains supplementary material available at 10.1007/s10926-024-10262-3.

## Introduction

Mental health conditions have a profound impact on individuals, their families, employers, and society worldwide [1, 2]. In Germany, the 12-month prevalence for any mental disorder is 27.8% in the general population, with the highest rate in the working-age population (26–36%) [3, 4]. Additionally, common mental disorders (CMDs), such as depression, anxiety, and adjustment disorders, have become the leading cause of long-term sickness absence days and durations in recent years [5, 6].

After being on long-term sickness absence and receiving inpatient treatment for CMDs, returning to work (RTW) is a complex and comprehensive process, in particular at the intersection between the mental healthcare system and the workplace–with several stakeholders involved [7, 8]. Besides the returning employee, these stakeholders include, for example, accompanying rehabilitation professionals and other treatment providers from the onset of sickness absence or treatment, occupational health physicians as the *‘bridge’* between both systems, and direct supervisors and RTW coordinators at the workplace [7–10].

In line with Young et al. [11], we view RTW as developmental and evolving process with four key phases: *off work*, *re-entry*, *maintenance*, and *advancement*. Many studies over the past decades have addressed work ability as predictor of RTW and mainly focused on the phases from *off-work* to *re-entry* examining factors that facilitate, hinder, or shorten the time to RTW due to CMDs [12–16]. In contrast, comprehensive knowledge about work participation and at-work outcomes during and after RTW is lacking [17, 18], while a better understanding of the employees’ experiences and needs to keep working is required [19]. For both returning employees and accompanying key RTW stakeholders, restoring and maintaining work ability during RTW is essential to enable sustained work participation. The present study covers all four RTW phases, from the end of inpatient treatment to 30 months later, and focuses especially on employees’ work ability experiences in the later RTW phases, i.e. *maintenance* and *advancement*.

To date, little research has examined the concept of work ability during the later RTW phases among employees with CMDs, although restoring and maintaining the ability to work are core objectives of RTW (e.g. also compared to the amount of existing literature in populations with musculoskeletal disorders [20–22]). This is somewhat surprising as self-reported lower work ability is associated with long-term sickness absences and potentially negative consequences like job loss, permanent work disability, or disability pensions [23, 24]. Moreover, restoring work ability and related resources, such as self-efficacy, can positively affect the achievement of a sustainable RTW [9, 14, 18, 25]. Regarding age and work ability, studies among different populations have shown that work ability decreases with age [26–28], but evidence on stable high trajectories of work ability over 16 years from mid-life towards retirement also exists [29]. Regarding sex and work ability, a recent representative employee cohort study showed no differences in sex and baseline work ability [30].

To better understand how to restore and maintain employees’ work ability during RTW with CMDs, both quantitative and qualitative research is needed. The overarching goal of this mixed methods follow-up study is to expand the understanding of restoring and maintaining work ability during the RTW process with CMDs. In our convergent mixed methods study, three research questions are addressed with a quantitative data strand, a qualitative data strand and lastly an integration of both data strands:

1. Do employees’ work ability ratings change during the 30-month follow-up? And do the work ability ratings differ regarding sex and age?
2. How do employees experience their work ability during their RTW process with CMDs at 6 and 12 months?
3. Do the quantitative health- and work-related survey data relate to the qualitative findings at 6 and 12 months?

## Methods

### Study Design and Setting

The present mixed methods follow-up study used a convergent parallel mixed methods design [31] and consisted of two data strands: a quantitative prospective cohort study [16, 32] and a qualitative narrative interview study [9, 33]. First, data collection and analysis of both data strands was done independently and in parallel, as both data strands had their unique research questions and analysis procedures [31]. Data analysis and data integration on the topic of work ability takes place with the complete study sample. The mixed methods follow-up study was conducted in the German mental healthcare system. Germany has a complex and fragmented mental healthcare system, with various service providers, settings, funding institutions, and pathways into treatment. More information on the German mental healthcare system and on the German sickness absence compensation system is provided in Sikora et al. 2022 [16] and 2019 [32], respectively. Regarding the timely preparation of RTW accommodations already during treatment, some actions are eventually taken by the clinicians, mainly to prepare a gradual RTW [16], but there is little contact between the healthcare system and the workplace setting. All employers in Germany are legally obliged to offer their employees, who have been on long-term sickness absence for more than six weeks within the last 12 months, an Operational Integration Management (OIM) programme. In the workplace setting, OIM can provide a structured framework in which the RTW process is professionally accompanied and RTW work accommodations can be implemented.

Regarding the outcome of interest and feasibility, eligible study participants were recruited from five cooperating clinics (two psychiatric clinics and three medical psychosomatic rehabilitation facilities) during their inpatient psychiatric or medical rehabilitation treatment for CMDs between August 2016 and November 2017. Inclusion criteria were: age between 18 and 60 years, previous sickness absence duration no longer than 6 months within the last 12 months (to avoid the inclusion of more chronic, severe conditions), part- or full-time employment, permanent or fixed-term employment for at least 18 months, intention of RTW with the present employer, and treatment of a first medical diagnosis and maximally one further diagnosis of: a depressive disorder (F32.0, F32.1, F32.2), a recurrent depressive disorder (F33.0, F33.1, F33.2), agoraphobia with a panic disorder (F40.01), a panic disorder (F41.0), a generalised anxiety disorder (F41.1), a mixed anxiety and depressive disorder (F41.2), or an adjustment disorder (F43.2). Ethical approval for the study was granted by the Hannover Medical School (MHH) Ethics Committee (ID: 3211–2016). All participants provided written consent.

### Study Samples

#### Quantitative Cohort Study

In the quantitative cohort study, four telephone measurements were conducted, starting in the last week before clinical discharge (T0), and then 6 (T1), 12 (T2), and 18 (T3) months later. An additional online survey was conducted 30 months after clinical discharge (T4). Quantitative data collection took place between August 2016 and May 2020. For the present analysis, *N* = 286 participants took part in the T0 baseline measurement, *N* = 269 participants (94.1%) in the T1 survey, *N* = 266 participants (93.0%) in the T2 survey, and *N* = 259 participants (90.6%) completed all four telephone measurements (T0–T3). For the additional online survey 30 months after baseline (T4), only the interested participants from the last telephone survey (T3) were invited (*N* = 238). A total of *N* = 157 participants (54.9% of the baseline sample) completed all five measurements (T0–T4).

#### Qualitative Sub-sample of the Cohort Study

In the qualitative sub-sample, three narrative interviews concerning the employees’ experiences during the RTW process were conducted, each with an average duration of 30 to 45 min, starting in the last week before clinical discharge at the clinic (T0), and then 6 (T1) and 12 (T2) months later via telephone. Qualitative data collection took place between August 2016 and March 2018. The qualitative sub-sample was selected by the quantitative study team according to the following sampling method, which was defined a priori in relation to the study goals: (1) additional interview interest and informed consent, (2) sex (equal proportion of women and men), (3) RTW expectation (75% positive vs. 25% negative), and (4) first diagnosis (50% with depression, 25% with anxiety disorder and 25% with adjustment disorder), see Sikora et al. [32] for more details. For the present analysis, all follow-up interviews from T1 (*N* = 32) and T2 (*N* = 30) were used jointly with the respective work ability ratings from the quantitative surveys.

### Measures

#### Work Ability

Quantitative and qualitative data collection on work ability was conducted independently and in parallel at nearly the same measurement points. Qualitative researchers were not informed about the quantitative work ability ratings before the interviews.

#### Quantitative Work Ability Ratings

At each measurement point (T0-T4), work ability was measured with the validated first item of the Work Ability Index, the Work Ability Score (WAS), to assess *‘the current work ability compared with its lifetime best’* [26]. The WAS is regarded as a reliable and valid measure to assess work ability, e.g. among workers on sickness absence with chronic musculoskeletal pain [34]. Comparative studies of the WAS and WAI have shown that the WAS is sufficiently valid and feasible to measure the status and progress of work ability in employee surveys, occupational health research, and for study populations on long-term sickness absence [24, 35–37]. Scores range from 0 *(‘completely unable to work’*) to 10 *(‘work ability at its best’*), with higher scores indicating a better work ability.

#### Qualitative Work Ability Experiences

In both narrative follow-up interviews (T1–T2), the following question was included at the end: “*How do you assess your current work ability and what, in your view, has contributed to this?”*. Each interview was externally audio recorded with the consent of the interviewees, transcribed, and saved pseudonymously [9, 33].

#### Health- and Work-related Data

Based on the existing RTW literature, key health- and work-related variables were used in the integrative data analysis: *self-rated health*, *depressive symptoms*, *work-privacy conflict*, *RTW self-efficacy*, *functional ability*, *social support*, *sense of community*, and *number of RTW accommodations*. Detailed information about the operationalisation and measurement of the health- and work-related variables is provided in Supplemental Material A.

### Data Analysis

**Fig. 1:**
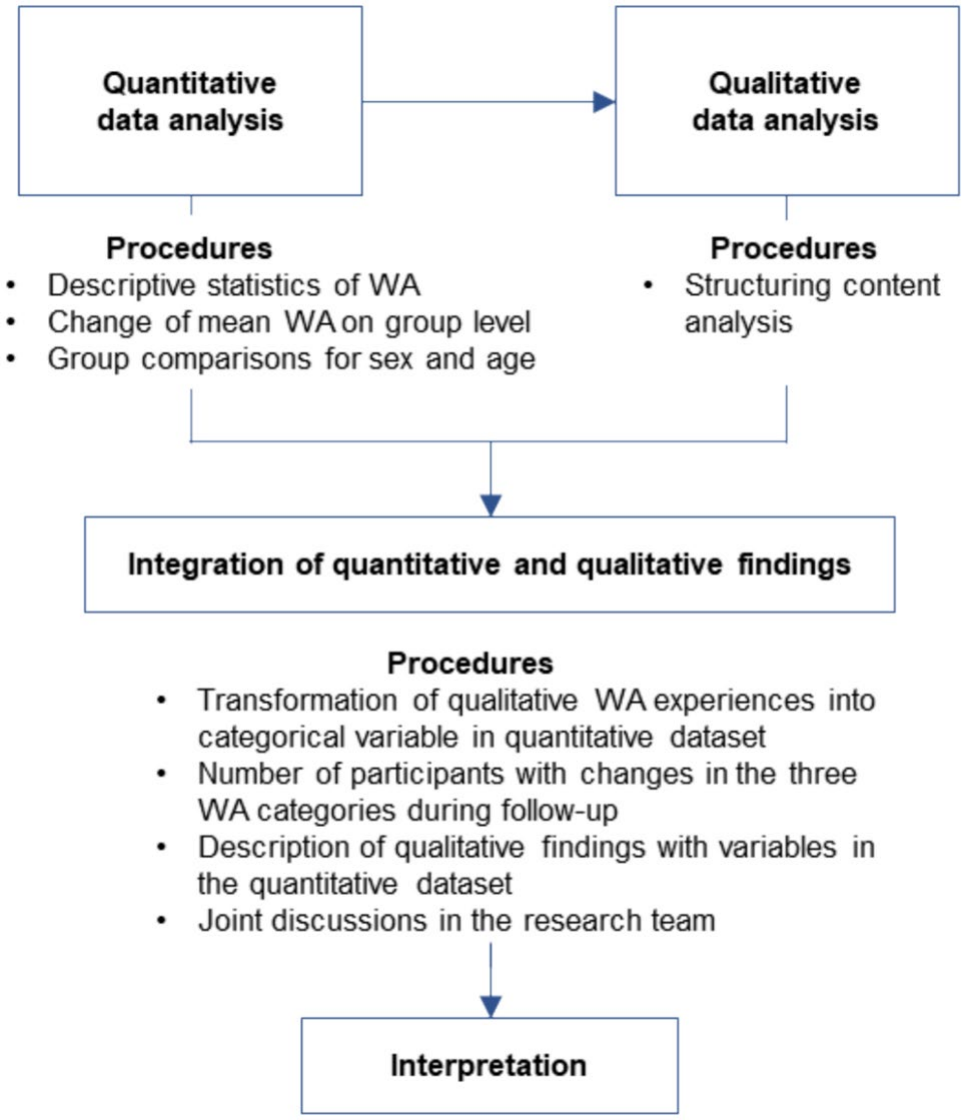
Schematic overview of the procedures of the three data analysis sections in the mixed methods study (WA = work ability)

Within a convergent parallel mixed methods study design, the quantitative and qualitative data will be first analysed separately, and then integrated [31]. After the quantitative data analysis and at the beginning of the qualitative data analysis, we linked the qualitative work ability interview passages from T1 and T2 with their respective quantitative work ability ratings from T1 and T2 (as illustrated by the arrow in Fig. 1). Finally, the integrative data analysis of quantitative and qualitative findings took place. Figure 1 provides the procedures of the two separate and the integrative data analysis parts.

#### Quantitative Data Analysis

Beyond descriptive statistics of work ability for the total sample, paired-samples t-tests (after checking that all assumptions were met) were used to compare the mean work ability ratings, assessed with the Work Ability Score (WAS, 0–10) between all measurement points from baseline to 30 months of follow-up. At each measurement point, work ability ratings were compared for sex and age (18–49 years vs. 50–60 years) differences using independent-samples t-tests. The WAS was used as continuous variable in all statistical analyses [37].

#### Qualitative Data Analysis

The qualitative data of the present study regarding the main category of work ability were analysed using the structuring qualitative content analysis according to Kuckartz and Rädiker [38]. Within an iterative analysis process, interview material is analysed systematically and methodologically controlled. In the first step, for the *N* = 32 participants of the qualitative sub-sample, the respective quantitative work ability ratings from the telephone surveys (T1 and T2) were linked to the follow-up interviews at T1 and T2 (see Supplementary Figure B). Due to the partly very small group sizes in each WAS response (0 to 10) and a largely balanced distribution of the number of interviews in each WAS response between T1 and T2 (see Supplementary Material Figure C), we did not further distinguish between T1 and T2 interviews and refer to ‘follow-up interviews’ covering both measurements. The pseudonymised interview passages regarding the interview question about work ability of all follow-up interviews (*N* = 62) were sorted in tabular form according to their respective quantitative work ability ratings from 0 to 10.

In the second step, the respective work ability descriptions were abstracted and compared with each other. On the basis of the summarised and abstracted work ability descriptions, the following three sub-categories, further referred to as themes, were inductively developed: ‘impact on everyday life’, ‘impact on working life’, and ‘contributions on how to restore/maintain work ability’. The work ability descriptions were systematised according to these themes. Then, based on the three themes, similarities and differences were identified between all WAS responses from 0 to 10. We noticed that the work ability descriptions between some responses of the WAS were more similar than between others. For example, we recognised a large difference in the description of the work ability experiences between WAS response 3 and WAS response 4, and between WAS response 6 and WAS response 7.

In the third step, three work ability experience groups were qualitatively identified and summarised. Similarities and differences between the three groups were elaborated, discussed and written down. In the last step, we visualised and summarised the qualitative results in joint displays as the first part of the integrative data analysis. During the structuring content analysis procedure, the research team met seven times (for one to two hours each time) for an in-depth discussion of the analysis and documentation of the results.

#### Integrative Data Analysis

**Fig. 2:**
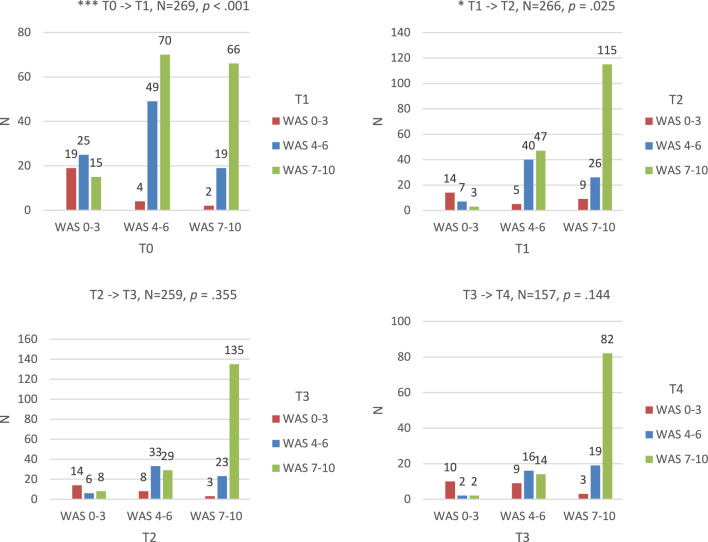
Number of participants in the three WAS categories (poor WAS: 0–3, moderate WAS: 4–6, good to very good WAS: 7–10) and the respective number of participants with changes between two measurement points

Based on the qualitative findings of the work ability experiences, we transformed the continuous work ability ratings of the WAS into a categorical variable with three categories (1: WAS 0–3, 2: WAS 4–6, 3: WAS 7–10). As shown in Fig. 2, we computed the number of participants with changes in the three WAS categories during follow-up from using McNemar Bowker tests. To *‘validate’* in the meaning of the mixed methods-based approach according to Creswell & Plano Clark [31], the qualitative findings were described in terms of quantitative health- and work-related variables. For each qualitative theme, key quantitative health- and work-related variables, measured at T1 and T2, were identified based on the existing RTW literature [32] and the qualitative results, and compared with the qualitative results. For the qualitative theme ‘impact on everyday life’, the variables *self-rated health*, *depressive symptoms*, and *work-privacy conflict* were used. For the qualitative theme ‘impact on working life’, the variables *RTW self-efficacy* and *functional ability* were used. For the qualitative theme ‘contributions on how to restore/maintain work ability’, the variables *social support, sense of community*, and *number of RTW accommodations* were used. In the last step of the integrative data analysis, differences between the three WAS categories were assessed with a chi-square test for categorical variables, and a one-way analysis of variance for continuous variables.

## Results

### Quantitative Work Ability Ratings During the RTW Process

Table 1 shows the mean work ability ratings for the total sample, and stratified by sex and age at all measurement points. The average work ability ratings of the employees increased on a group level from T0 (*M* = 5.33, *SD* = 2.19, *N* = 269) to T1 (*M* = 6.35, *SD* = 2.03, *N* = 269), *t* (268) = − 7.82, *p* < .001 (two-tailed) and between T1 (*M* = 6.37, *SD* = 2.01, *N* = 259) and T3 (*M* = 6.73, *SD* = 2.12, *N* = 259), *t* (258) = − 2.62, *p* = .009 (two-tailed).

**Table 1:** Quantitative work ability ratings at each measurement point for the quantitative study cohort, and stratified by sex and age

	WAS												
	Total sample			Women			Men			*t*	*p*	mean difference	95% confidence interval
	*N*	*M*	*SD*	*N*	*M*	*SD*	*N*	*M*	*SD*				
T0	286	5.27	2.16	133	5.39	2.32	153	5.17	2.02	0.862	.389	.221	− .283 to .726
T1	269	6.35***	2.03	126	6.40	2.17	143	6.29	1.90	0.447	.655	.111	− .378 to .600
T2	266	6.56	2.14	124	6.60	2.27	142	6.52	2.02	0.318	.751	.084	− .435 to .602
T3	259	6.73**	2.12	122	6.89	2.10	137	6.58	2.14	1.172	.242	.310	− .211 to .830
T4	157	6.48	2.15	72	6.71	2.05	85	6.28	2.23	1.239	.217	.426	− .253 to 1.105

				18–49 years			50–60 years			*t*	*p*	mean difference	95% confidence interval
				*N*	*M*	*SD*	*N*	*M*	*SD*				
T0				137	5.22	2.08	149	5.32	2.24	− 0.403	.687	− .103	− .607 to .401
T1				124	6.35	1.83	145	6.34	2.19	0.008	.994	.002	− .488 to .492
T2				122	6.75	1.83	144	6.40	2.36	1.393	.165	.358	− .148 to .865
T3				120	7.00	1.86	139	6.50	2.31	1.943	.053	.504	− .007 to 1.014
T4				73	6.88	2.06	84	6.13	2.18	2.194	.030*	.746	.074 to 1.417

***Statistically significant different from prior T0 measurement (*p* < .001)

**Different from T1 measurement (*p* = .009)

*p < 0.05; (WAS = Work Ability Score)

No differences were found in the mean work ability ratings for women and men (see Table 1). The highest mean work ability rating (*M* = 7.00, *SD* = 1.86) was found in the age group of 18–49 years at T3. At T4, a small difference in the mean work ability ratings was found between the two age groups (18–49 years (*M* = 6.88, *SD* = 2.06) *vs.* 50–60 years (*M* = 6.13, *SD* = 2.18)).

### Qualitative Work Ability Experiences During the RTW Process

During the structuring content analysis of the *N* = 62 qualitative follow-up interviews, three work ability experiences during the RTW process with CMDs emerged, labelled: *poor*, *moderate*, and *good to very good work ability experience*. The interview content was structured along the following three themes: ‘impact on everyday life’, ‘impact on working life’, and ‘contributions on how to restore/maintain work ability’. A descriptive summary on each theme and each work ability experience is provided in Tables 2–4.

**Table 2:** Joint display of results from the qualitative follow-up interviews on the theme ‘impact on everyday life’ with health- and work-related variables, measured at T1 and T2

	Qualitative work ability experiences		
	Poor work ability (0–3) (T1 *N* = 6; T2 *N* = 3)^a^	Moderate work ability (4–6) (T1 *N* = 10; T2 *N* = 7)^a^	Good to very good work ability (7–10) (T1 *N* = 16; T2 *N* = 20)^a^
Impact on everyday life ^b^	*- huge physical and/or psychological problems that led to major restrictions in everyday life* *- everyday skills did not yet work well (or at all) (e.g., low self-efficacy and low stress tolerance, often together with severe concentration and memory problems)* *- emotional instability, inner restlessness, tension, and/or rapid exhaustion*	*- on the one hand, a more or less ‘ordinary’ ability to work, progress in stabilisation, and a return to ‘normality’, but* *- on the other hand, not yet found a stable balance in everyday (work) life again due to daily form dependency of the work ability (see next line)* *- imbalance of work and private life (again)—a lot of energy goes into work to the cost of private life (too tired after work, and no energy to reconcile other commitments and leisure time)*	*- found a good balance between work and private life again; succeed very well in detaching from work, recovering and enjoying life (and work) again* *-* *actively shaping and rediscovering family life, life with partners, children and/or friends to draw meaning, joy, and energy for everyday life from it* *- activation of old or new hobbies as exclusive time for oneself*
T1 Good self-rated health, % (n)***	16.7 (4)	66.7 (62)	93.4 (141)
T2 Good self-rated health, % (n)***	14.3 (4)	68.1 (49)	91.5 (151)
T1 Depressive symptoms (0–24), Mean ± SD (n)***	13.28 ± 5.86 (25)	9.37 ± 4.35 (93)	5.40 ± 3.15 (151)
T2 Depressive symptoms (0–24), Mean ± SD (n)***	14.25 ± 5.35 (28)	9.99 ± 4.17 (73)	5.61 ± 3.49 (172)
T1 Work-privacy conflict (0–100), Mean ± SD (n)***	62.50 ± 25.25 (14)	50.89 ± 21.34 (84)	38.48 ± 23.65 (148)
T2 Work-privacy conflict (0–100), Mean ± SD (n)***	61.88 ± 23.58 (16)	53.31 ± 25.86 (71)	38.40 ± 23.51 (153)

^a^ Of the qualitative follow-up interviews (*N* = 62)

^b^ Qualitative theme

*** *p* < .001 (differences between the three WAS categories)

**Table 3:** Joint display of results from the qualitative follow-up interviews on the theme ‘impact on working life’ with health- and work-related variables, measured at T1 and T2

	Qualitative work ability experiences		
	Poor work ability (0–3) (T1 *N* = 6; T2 *N* = 3)^a^	Moderate work ability (4–6) (T1 *N* = 10; T2 *N* = 7)^a^	Good to very good work ability (7–10) (T1 *N* = 16; T2 *N* = 20)^a^
Impact on working life ^b^	*- so many everyday life difficulties that work has not even been thought of yet, or would have led to direct mental overload* *- thoughts regarding RTW associated with fears and concerns, little self-confidence; RTW mostly seemed impossible at that stage*	*- daily form dependency of the work ability with difficulties in concentration and exhaustion at work from time to time, noticeable stress limits, especially when unforeseen things happen, shortly before holidays or when the daily rhythm is disrupted again (e.g., due to an illness)* *- imbalance of work and private life (again): private problems have a negative impact at work (or *vice versa*)*	*- can enjoy working life again* *- remain mindful with oneself and work, even under pressure* *- learned how to create a sufficient distance between inner demands and external requirements, and how to deal sensitively with stress limits and/or conflicts at work (and in private life)* *- improvements in private life also have a positive impact on working life and work ability*
T1 RTW self-efficacy (1–6), Mean ± SD (n)***	3.08 ± 1.15 (25)	4.31 ± 0.72 (93)	4.98 ± 0.59 (151)
T2 RTW self-efficacy (1–6), Mean ± SD (n)***	3.17 ± 1.25 (27)	4.25 ± 0.73 (73)	4.88 ± 0.57 (165)
T1 Functional ability: Managing (1–5), Mean ± SD (n)***	3.01 ± 0.69 (25)	2.43 ± 0.57 (93)	1.78 ± 0.47 (151)
T2 Functional ability: Managing (1–5), Mean ± SD (n)***	3.17 ± 0.50 (28)	2.43 ± 0.59 (73)	1.83 ± 0.58 (165)
T1 Functional ability: Cooperation/Communication (1–5), Mean ± SD (n)***	2.54 ± 0.85 (25)	1.92 ± 0.59 (93)	1.48 ± 0.47 (151)
T2 Functional ability: Cooperation/Communication (1–5), Mean ± SD (n)***	2.28 ± 0.81 (28)	1.88 ± 0.65 (73)	1.52 ± 0.47 (165)

^a^ Of the qualitative follow-up interviews (*N* = 62)

^b^ Qualitative theme

*** *p* < .001 (differences between the three WAS categories)

**Table 4:** Joint display of results from the qualitative follow-up interviews on the theme ‘contributions on how to restore/maintain work ability’ with health- and work-related variables, measured at T1 and T2

	Qualitative work ability experiences		
	Poor work ability (0–3) (T1 *N* = 6; T2 *N* = 3)^a^	Moderate work ability (4–6) (T1 *N* = 10; T2 *N* = 7)^a^	Good to very good work ability (7–10) (T1 *N* = 16; T2 *N* = 20)^a^
Contributions on how to restore/maintain work ability^b^	*- none described*	*- mainly individual aspects, e.g., learned techniques for stress reduction (especially relaxation and breathing exercises), setting and naming boundaries, group and/or individual psychotherapy, support with medication, private compensation, or resolution of private problems* *- some (mostly individual) work accommodations: less overtime and work pressure set for oneself, flexible division of tasks, and no blaming for when something has not been achieved;* *- support of co-workers*	*- similar individual aspects and individual work accommodations as Group 2, plus:* *- changes in attitude, behaviour and actions that led to an inner calm/peace (e.g., where over 100% work ability is no longer the goal) in order not to overstrain oneself again,* *- permanent reduction in working hours planned or already implemented,* *- safe and supportive work environment,* *- flexibility in the performance of tasks,* *- less pressure from work environment,* *- consistent use of breaks and holidays,* *- change of work, courage to start something new, and* *- mostly an interplay of individual and work accommodations together*
T1 Social support colleagues (0–100), Mean ± SD (n)	58.93 ± 25.68 (14)	62.50 ± 23.51 (83)	69.05 ± 21.91 (147)
T2 Social support colleagues (0–100), Mean ± SD (n)***	60.83 ± 19.40 (15)	57.39 ± 20.11 (71)	69.33 ± 19.82 (152)
T1 Sense of community (0–100), Mean ± SD (n)**	68.45 ± 17.04 (14)	67.97 ± 18.75 (83)	76.73 ± 17.46 (147)
T2 Sense of community (0–100), Mean ± SD (n)***	62.78 ± 16.02 (15)	65.96 ± 18.83 (71)	75.22 ± 16.22 (153)
T1 ≥ 1 RTW work accommodation, % (n)**	8.0 (2)	31.2 (29)	42.4 (64)
T2 ≥ 1 RTW work accommodation, % (n)	7.1 (2)	5.5 (4)	9.1 (15)

^a^ Of the qualitative follow-up interviews (*N* = 62)

^b^ Qualitative theme

*** *p* < .001

** *p* < .01 (differences between the three WAS categories)

#### Poor Work Ability Experience

All participants with work ability ratings between 0 and 3 at T1 and T2 were still incapacitated for work and returned to work only sporadically until T2. Some participants were about to return to work. The participants described their current work ability as *‘very bad, ‘insufficient’, ‘against zero’,* and/or felt *‘not able to work’*. The main focus was on coping with their illness(es), dealing with everyday life, solving additional problems (such as financial debts, partner violence, addiction in the family), and restoring their health. For most participants addressing the issue of *‘work-induced’* stress, almost no resources were available yet for dealing with work and RTW.

*“At the moment, it [the work ability] is close to zero. Just that stress that would come at me at work, I wouldn't be able to get through that at all at the moment. It would probably go well for three to four weeks, and then I would have put myself under such a stress level that I would probably collapse again, because it is simply physical and mental stress at work as well. ... At the moment I wouldn't have anything to say against it: ‘Good, I can handle it.’” (interview A1, T1)*

*“...but I’m not yet ready to concentrate, for example, and I don’t know whether I’m ready in terms of perception (...) to have everyone in view yet. So that’s what’s keeping me busy.” (interview A2, T1)*

*“Because I’m not fit for work at the moment, ... I hardly sleep, I can’t concentrate, I forget everything, my head is constantly under power, so to speak, ... I’m always ruminating, I don’t know how things will go on, it’s all a great burden to me. And the social withdrawal is also a problem. I also have panic attacks...although at some point I said I’d just go back to work and everything would be fine, right? But it really doesn’t work.” (interview A3, T2)*

#### Moderate Work Ability Experience

Most participants with a work ability rating between 4 and 6 at T1 and T2 had returned to work and described their work ability on average as predominantly *‘ordinary’, ‘normal’, ‘quite ok’,* and/or *‘depending on the day’*. They managed their work and everyday life well, except when unforeseen things happened or when new emotionally stressful situations arose—in professional or private life. Sometimes, their work ability quickly changed again depending on the daily form. These participants perceived themselves as emotionally stable as long as the work demands remained manageable. If the work demands were no longer manageable, the continuous stabilisation and improvement of their work ability was at risk, which made their work ability fragile. The participants hardly mentioned work accommodations beyond individual aspects, such as not putting so much pressure on yourself at work and working less overtime, during RTW to support the improvement of the work ability and the sustainability of their RTW.

*“So I continue to work and work a lot, but it’s too much of a burden on my private life.... And I’m fit for work, yes, but maybe still seventy percent. So at the moment the balance is not so good, because I invest a lot of energy in work and come home and I’m very tired and then I just often don’t have the strength to reconcile the other things; obligations, but also free time.” (interview A4, T1)*

*“Yes, there are also differences: there are days when I think you’re back to your old self, and then there’s another hour when that’s not the case. But I already have the feeling that these hours, when I think I’m the same, are becoming more frequent again.” (interview A5, T1)*

*“I realise that my work is demanding and I have to be very concentrated; not with everything, but with many things. And if I am distracted mentally or if it puts a lot of stress on me, then it is difficult to be there with full attention. On the other hand, I can arrange it a little bit. ...And the fact that I don't work so much overtime any more is good for me. ...But yes, of course, when I’m not doing so well and that depends on my daughter, when things are shaky, or also with my husband, who wasn’t doing so well at times, I definitely notice that at work.” (interview A6, T1)*

*“Yes, my ability to work is rather satisfactory to below average. The fact that I was sick for a long time and it was difficult to get back into a normal rhythm... and mentally I am now also, I would say, rather average, but in a better mood (than 2 years ago), ... so in this respect I have aids, I know how to deal with the negative things, but they don't get any better, do they? No, you just stop them from getting worse.” (interview A7, T2)*

#### Good to Very Good Work Ability Experience

All participants with a work ability rating between 7 and 10 at T1 and T2 had returned to work, predominantly enjoyed their work, and generally found a new way to cope with their work and everyday life. The participants described their current work ability as *‘good’, ‘very good’, ‘relatively high’,* and/or *‘fully functioning’*. They established a stable work ability and stress resistance, and did not seem negatively influenced or thrown off course by unforeseen events. Moreover, they appeared to succeed well in controlling and favourably influencing themselves and their environment regarding their (mental) health. Factors described as negatively affecting their work ability were an increased workload without any flexibility, and age. Central to their good to very good work ability seemed to be the experience of their increasingly stabilising self-efficacy. During RTW, the participants experienced and learned that they can again master even challenging situations. This experience created trust in themselves, the work team, the work environment, and space for sustainable change.

*“Yes, well, I rate my ability to work relatively highly. And today too, we had a stressful day, from my point of view, but what was also confirmed, and yesterday, too, and I just stayed very calm with this stress. Otherwise, I would have been more jittery and run with it, but I was able to block that out and said: ‘No, it is useless’. ... And the whole work environment, it's a great environment, and I just feel safe and secure.” (interview A8, T1)*

*“I would rate my ability to work as good at the moment, it has improved because I had a conversation with my boss and tried to reduce this workload a bit, to distribute things, to allow myself more holidays and simply not to be under so much pressure anymore, how can I manage this whole workload? Instead, I work through one thing after another and then move on to the next. Of course, there are always projects that come up that you haven't planned for, but nevertheless I try not to work so much overtime again; and to really take my holidays consistently, just to switch off again; to re-charge my batteries for the next tasks”. (interview A9, T2)*

*“…And also, of course, the goodwill of the colleagues, the open discussions with colleagues, not with every colleague, but that I don’t have to keep it quiet like that anymore, but if someone has a problem with it, then he or she should have the problem, but the people who know what that means, with whom the contact has actually become better and that is also beneficial for my work.” (interview A10, T2)*

### Integration of the Quantitative and Qualitative Findings

Based on the qualitative work ability experiences, the continuous work ability ratings of the quantitative study cohort were transformed into a categorical variable with three categories (poor WAS: 0–3, moderate WAS: 4–6, good to very good WAS: 7–10). Figure 2 shows the number of participants in the three WAS categories and the number of participants with changes between the three categories from one measurement to another during follow-up. For example, out of *N* = 59 employees with a poor WAS (0–3) at T0, at T1 measurement *N* = 19 still perceived their work ability as poor (0–3), *N* = 25 perceived their work ability as moderate (4–6), and *N* = 15 perceived their work ability as good to very good (7–10) (see the upper left corner in Fig. 2). Significant changes were found between T0–T1 and T1–T2.

#### Support of Qualitative Findings by Quantitative Health- and Work-related Variables

The joint displays in Tables 2–4 show differences for almost all health- and work-related variables. For the qualitative theme ‘impact on everyday life’, where mostly physical and mental health-related aspects and challenges were described, employees who experienced a poor work ability at T1 and T2 also rated their health mainly as ‘poor’, reported high depressive symptoms (mean PHQ-8 > 10 at both measurements), and the highest work-privacy conflicts (this question was only answered by those who made an RTW attempt) (Table 2). As shown in Table 3, for the qualitative theme ‘impact on working life’, employees who experienced a good to very good work ability at T1 and T2 also rated their RTW self-efficacy and their work-related functional ability as highest in the study cohort. For the qualitative theme ‘contributions on how to restore/maintain work ability’, employees with poor work ability experiences could hardly name any concrete measures to improve their work ability, as reflected in the frequencies of RTW accommodations (Table 4). Most RTW accommodations were used until T1 measurement. Concerning the perceived social support of colleagues at T1 measurement, no differences between the three WAS categories were found.

## Discussion

In the present mixed methods follow-up study, employees’ work ability increased between 0 to 6 months, i.e. in the earlier RTW phases from *off-work* to *re-entry*, and between 6 to 18 months, i.e. in the later RTW phases *maintenance* and *advancement*. No sex differences and only a small age difference was found 30 months later. Three qualitative work ability experience groups emerged. Employees with good to very good work ability experience (WAS 7–10) comprised the majority of the total study sample after the initial re-entry phase and described qualitatively the most contributions on how to restore and maintain their work ability. Employees with a moderate work ability experience (WAS 4–6) mostly returned to work, but reported fewer work accommodations to restore and maintain their work ability, which may hindering them to develop into the maintenance and advancement RTW phases. Employees with a poor work ability experience (WAS 0–3) need to first restore their health situation, and then find a timely re-entry to work. The quantitative health- and work-related data supported the qualitative findings. Thereby, we expanded the understanding of work ability during the RTW process on several levels: with examining quantitative longitudinal data of employees’ work ability over 30 months and through the entire RTW process, by adding qualitative work ability experiences during the RTW process, and by integrating the quantitative and qualitative data.

To date, only a few studies have addressed work ability during the RTW process with CMDs, in particular over a longer follow-up period. The results of the current mixed methods follow-up study complement the previous results from the qualitative study strand by Schröder et al. [33], in which four types of coping strategies during RTW were identified: employees who are (1) insight-oriented, (2) utility-/benefit-oriented, (3) recovery-oriented, and (4) powerless-oriented with complex problem situations in several areas of their life [33]. These four types of coping strategies may now be further extended by their respective work ability experience. It can be assumed that the insight-oriented participants would also be the participants with the good to very good work ability, the utility-oriented and recovery-oriented participants with the more fragile moderate work ability, and the powerless-oriented participants with the poorest work ability.

The findings of Danielsson et al. [39], who described in a qualitative study different strategies to keep working with CMDs, the descriptions of their category ‘reflexive adaption’, and related sub-categories like ‘reconsidering one’s attitude to work’, ‘modifying the work frame’, and ‘reaching for managerial and collegial support’ greatly overlap with our findings of the employees with a good to very good work ability. Participants in the present study and those in the study of Danielsson et al. seem to have acquired a higher level of reflectiveness regarding their work and private life, together with necessary changes in attitude, and realised individual and work accommodations to promote a sustainable RTW and work participation.

In a recent qualitative study, Nielsen and Yarker [40] explored experiences of employees’ post RTW journeys as well as barriers and facilitators ‘to stay and thrive at work’ again. During the first four months post RTW, Nielsen and Yarker [40] identified three qualitative post RTW trajectories: thrivers, survivors, and exiteers. Although the study period was shorter, and work ability was not directly addressed, the narrative descriptions of three post RTW trajectories seem to have great overlap with the employees experiencing moderate and good to very good work ability in the present study. For example, descriptions like ‘…*are still experiencing ups and downs by month 4*’, *‘…still is thrown off kilter easily: “It’s off and on”*’ from the groups of survivors [40] are very similar to descriptions of employees experiencing moderate work ability. The examples of the exiteers group seem to correspond only partially with the work ability experiences of our sample. Among the employees experiencing poor work ability, primarily no return (or only attempts to return) was observed and the first priority was to stabilise their health situation before RTW can even be planned. Nevertheless, both exiteers and employees with poor work ability seem to have the fewest resources in their RTW process or have lost far more resources than other employees on their way into the mental health crisis. Our mixed methods analysis also indicated that better work ability is related to increased resources, with the (re)acquisition and use of existing and additional individual, social and work-related resources. In our previous qualitative study [9], participants described and experienced the path into the mental health crisis as a gradual process and, in this sense, as a spiral of resource loss. Later, during RTW, the participants described a sustainable RTW primarily as a way of using existing and new resources in the sense of a resource gain spiral [9, 41].

In the present study, the WAS was applied among employees with CMDs during their RTW process. Although our categorisation of work ability as *poor*, *moderate,* and *good to very good* is comparable with Gould et al. [28], the score ranges for each group are different. If we had used Gould’s categorisation [28], we might have underestimated the work ability of the employees during their RTW process. Hence, it is important to consider the study population, the administrative setting, and the categorisation’s original purpose.

### Strengths and Limitations

The strengths of this study are the convergent parallel mixed methods study design with the joint evaluation of both longitudinal quantitative and qualitative data embedded in a prospective longitudinal cohort study with a 30-month follow-up period. Our methodological approach of linking qualitative interview data with quantitative work ability ratings allowed us to jointly capture employees’ actual work ability experiences with their ratings during the RTW process. Mixed method *‘validation’* was enhanced through the joint discussions within the mixed methods research team. The data integration showed high conformity with the quantitative data and underlined the qualitative results.

A limitation of this study is that all data were self-reported. Since we did not draw a random sample in the quantitative study cohort, selection bias may have occurred. A healthy entrance effect due to our narrow study inclusion criteria cannot be excluded [16]. A possible recall bias may have occurred due to the partly retrospective data collection of RTW measures and work accommodations, which were assessed for the duration of the last six months. Furthermore, given the study design, cause-effect relationships cannot be deferred from the findings. As the present study was conducted in the German social security system, the translation of findings to other countries and jurisdictions should be done with caution.

### Implications

Our findings offer both practitioners and researchers a look beyond a solely quantitative assessment of work ability during RTW, and provide a more comprehensive understanding of work ability in the RTW process with CMDs. Occupational health professionals and other key RTW stakeholders may use the knowledge about the work ability ratings and experiences to better support ongoing and future RTW processes of employees with CMDs. Assessing the current work ability with the WAS in trusting RTW conversations can be a starting point and/or a continuous ‘check-in’ option for further dialogue and tailored individual support during RTW. Employees with *poor work ability* may need the most immediate healthcare support (e.g. intensified aftercare, outpatient psychotherapy), counselling services, and practical support to address their complex situation, and to restore their work ability. When their situation has stabilised, a timely RTW may be prepared and planned together with care. For employees with *moderate work ability* who did not yet find a stable balance in everyday (work) life again due to daily work ability fluctuations, RTW stakeholders may support this stabilisation in work life with the proactive design of work accommodations and working conditions. Regular feedback talks with supervisors may also help to find a new balance with work tasks and demands. For employees with *good to very good work ability*, RTW stakeholders can help to maintain this good level of work ability, when employees know about professional RTW stakeholders at the workplace, and have the possibility to seek their selective support when needed.

In Germany, regarding the better implementation of work accommodations during RTW, employees with long-term sickness absence of six weeks or more within the last 12 months shall receive an OIM offer from their employer. OIM is a legal obligation for employers in Germany to support the RTW process of their employees on long-term sickness absence. The main goal of OIM is to clarify the options for overcoming work incapacity, to prevent renewed work incapacity, and to maintain the workplace [32]. Unfortunately, OIM is still offered far too little, i.e. in the latest representative data collection 2018, to only about 40% of eligible employees [42]. Within a professional and trusted OIM process, tailored work accommodations to restore and maintain employees’ work ability can be identified and implemented.

## Conclusion

The present study provides new insights into different aspects of work ability experiences, especially during the later RTW phases, where restoring and maintaining work ability is essential for a sustained work participation. This knowledge may help RTW stakeholders to better tailor their support during the RTW process.

## Supplementary Information

Below is the link to the electronic supplementary material.

Supplementary file1 (DOCX 44 kb)

## Data Availability

The datasets analysed in the present study are available from the corresponding author on reasonable request after official permission by the privacy officer at the Federal Institute for Occupational Safety and Health.
