# Supplementary material for: Work Ability during the Return to Work Process: Results from a Mixed Methods Follow-Up Study Among Employees with Common Mental Disorders
Source: J Occup Rehabil. 2025 Feb 6;36(1):248–61. doi: 10.1007/s10926-024-10262-3 (PMC12906556; doi:10.1007/s10926-024-10262-3)
Supplement: Supplementary file 1 — Supplementary file1 (DOCX 44 kb) [file 10926_2024_10262_MOESM1_ESM.docx]

**Supplemental material A: Overview of the health- and work-related measures**

**Self-rated health** was measured with the question ‘How would you describe your current health?’ [1] on a 5-point Likert scale, and was dichotomised into ‘poor’ (poor, bad) versus ‘good’ (satisfactory, good, very good) [2].

**Depressive symptoms** were measured with the 8-item Patient Health Questionnaire [3]; scores range from 0 to 24, with higher scores indicating more depressive symptoms.

**Work-privacy conflict, social support from colleagues,** and **sense of community** were measured with the Copenhagen Psychosocial Questionnaire (COPSOQ) [4, 5]. COPSOQ scores range from 0 to 100, with higher scores indicating more work-privacy conflicts, but also more perceived social support and higher sense of community.

**RTW self-efficacy** was measured with a translated version of the questionnaire by Lagerveld et al. [6, 7]. RTW self-efficacy scores range from 1 to 6, with higher scores indicating higher perceived self-efficacy regarding RTW.

**Functional ability** was measured with two sub-scales (managing and cooperation/communication)

from the German version of the Norwegian Function Assessment Scale [8, 9]. Scores range from 1 to 5, with higher scores indicating more difficulties regarding the functional ability.

Implemented **RTW accommodations** were measured with a self-developed list derived from the work accommodation needs for RTW [6]. RTW accommodations were retrospectively for the last 6 months. A categorical variable with *‘one or more implemented RTW accommodation’* vs. *‘no implemented RTW accommodation’* was computed.

References:

1. SOEP Group. SOEP-Core—2015: personal questionnaire (with reference to variables). SOEP survey papers 419: Series A—survey instruments. Berlin; 2017 (in German).
2. Sampere M, Gimeno D, Serra C, Plana M, Lopez JC, Martinez JM, et al. Return to work expectations of workers on long-term non-work-related sick leave. J Occup Rehabil. 2012;22(1):15–26.
3. Kroenke K, Spitzer R. The PHQ-9: a new depression diagnostic and severity measure. Psychiatr Ann. 2002;32:509–515.
4. Nübling M, Stößel U, Hasselhorn H, Michaelis M, Hofmann F. Measuring psychological stress and strain at work: evaluation of the COPSOQ questionnaire in Germany. GMS Psychosoc Med. 2006;3:Doc05.
5. Pejtersen JH, Kristensen TS, Borg V, Bjorner JB. The second version of the Copenhagen psychosocial questionnaire. Scand J Public Health. 2010;38(3 Suppl):8–24.
6. Sikora A, Schneider G, Stegmann R, Wegewitz U. Returning to work after sickness absence due to common mental disorders: study design and baseline findings from an 18 months mixed methods follow-up study in Germany. BMC Public Health. 2019;19(1):1653.
7. Lagerveld SE, Blonk RWB, Brenninkmeijer V, Schaufeli WB. Return to work among employees with mental health problems: development and validation of a self-efficacy questionnaire. Work Stress. 2010;24(4):359–375.
8. Jankowiak S, Rose U, Kersten N. Application of the ICF based Norwegian function assessment scale to employees in Germany. J Occup Med Toxicol. 2018;13:3.
9. Østerås N, Brage S, Garratt A, Benth JS, Natvig B, Gulbrandsen P. Functional ability in a population: normative survey data and reliability for the ICF based Norwegian function assessment scale. BMC Public Health. 2007;7:278.

**Supplemental material B:**


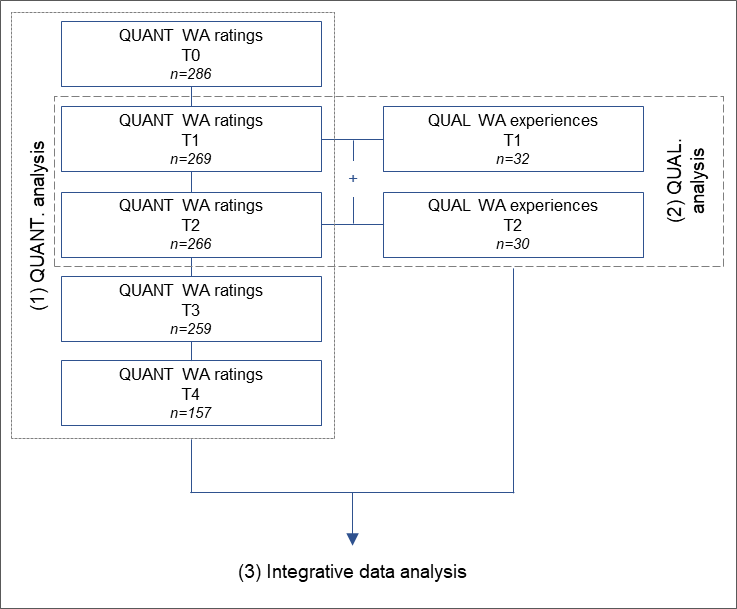


**Supplementary Figure B** Flow diagram of mixed methods data collection and analysis *(QUANT=quantitative, QUAL=qualitative, WA=work ability)*

**Supplemental material C:**

**Supplementary Figure C** Number of all N=62 T1 and T2 interviews linked to their quantitative work ability ratings (presented for each WAS response category) at T1 and T2 measurement
